# An Update on Nickel-Titanium Rotary Instruments in Endodontics: Mechanical Characteristics, Testing and Future Perspective—An Overview

**DOI:** 10.3390/bioengineering8120218

**Published:** 2021-12-16

**Authors:** Alessio Zanza, Maurilio D’Angelo, Rodolfo Reda, Gianluca Gambarini, Luca Testarelli, Dario Di Nardo

**Affiliations:** Department of Oral and Maxillofacial Sciences, Sapienza University of Rome, 00161 Rome, Italy; alessio.zanza@uniroma1.it (A.Z.); maurilio.dangelo@uniroma1.it (M.D.); gianluca.gambarini@uniroma1.it (G.G.); luca.testarelli@uniroma1.it (L.T.); dario.dinardo@uniroma1.it (D.D.N.)

**Keywords:** endodontics, endodontic rotary instruments, Nickel-Titanium alloy, root canal treatment

## Abstract

Since the introduction of Nickel-Titanium alloy as the material of choice for the manufacturing of endodontic rotary instruments, the success rate of the root canal therapies has been significantly increased. This success mainly arises from the properties of the Nickel-Titanium alloy: the biocompatibility, the superelasticity and the shape memory effect. Those characteristics have led to a reduction in time of endodontic treatments, a simplification of instrumentation procedures and an increase of predictability and effectiveness of endodontic treatments. Nevertheless, the intracanal separation of Nickel-Titanium rotary instruments is still a major concern of endodontists, with a consequent possible reduction in the outcome rate. As thoroughly demonstrated, the two main causes of intracanal separation of endodontic instruments are the cyclic fatigue and the torsional loads. As results, in order to reduce the percentage of intracanal separation research and manufacturers have been focused on the parameters that directly or indirectly influence mechanical properties of endodontic rotary instruments. This review describes the current state of the art regarding the Nickel-Titanium alloy in endodontics, the mechanical behavior of endodontic rotary instruments and the relative stresses acting on them during intracanal instrumentation, highlighting the limitation of the current literature.

## 1. Introduction

The history of Endodontics is characterized by two eras, divided each other by the introduction of Nickel-Titanium (NiTi) alloy as the most eligible material for the manufacturing of endodontic rotary instruments. Its introduction, in fact, has thoroughly changed the instrumentation procedures of endodontic root canal systems, so as to be considered as a technological revolution which established the beginning of the modern endodontics. The exact moment of this passage corresponds to the publishment by Walia et al. of the article titled “An initial investigation of the bending and torsional properties of Nitinol root canal files”, in which for the first time the Authors proposed the NiTi alloy as a material for the manufacturing of endodontic instruments, considering the great success that this alloy was having in orthodontics [1]. In this article the Authors highlighted the limitations of the instrumentation of root canal system with stainless-steel (SS) manual files, that could be summarized in the ledge or zip formation, the canal perforation or transportation and the separation of the instruments, especially in the endodontic treatment of canals with severe curvatures [2,3,4]. These complications mainly arise from the stiffness of the SS alloy, especially with the increase of the instrument diameter.

In the following years, several Authors proposed the use of NiTi instruments at high rotational speed with the use of engine-driven endodontic motors, by exploiting the mechanical properties of NiTi alloy, such as superelasticity and shape memory effect [5,6,7]. The technological revolution, with the innovations made by the production processes in the field of instruments manufacturing, in particular with regard to their dimensions and conicity, greatly facilitated the instrumentation procedures of root canals, improving their chemo-mechanical disinfection increasing the success rates of endodontic treatments [8].

In the light of the above, the introduction of NiTi in Endodontics has brought some undisputed advantages, summarized essentially in three points [7,9].

### 1.1. Advantages in Using NiTi Rotary Instruments

- Reduction in time of endodontic treatments

Instrumentation technique with manual SS files requires a larger number of tools and longer operating times. Instead, the increased cutting efficiency of NiTi rotary instruments and the use of increased taper instruments allow clinicians to improve these parameters of endodontic treatment [10,11,12].

- Simplification of instrumentation procedures

The special properties of the NiTi instruments have made it possible to considerably simplify the instrumentation technique compared to the traditional procedural steps carried out through the use of SS files [11,13,14]. Thanks to the better mechanical characteristics of rotary instruments than manual ones, it’s possible to shape the root canals respecting their original trajectories not altering their original anatomy [15,16].

- Increase of predictability and effectiveness of endodontic treatments

The superelasticity of NiTi alloy ensures the use of endodontic instruments with an increased taper without an excessive risk of fracture due to bending or cyclic fatigue, improving the process of root canal shaping and therefore of root canal filling [17]. For all these reasons, the success rates of endodontic treatments performed with NiTi rotary instruments is significantly greater than those performed with SS manual instruments [18,19,20,21,22].

### 1.2. Nickel-Titanium Alloy

The fundamental characteristics that allow endodontists and manufacturers of considering the NiTi as the material of choice for the production of endodontic rotary instruments reside in the intrinsic properties of this material.

The Nitinol was discovered in 1959 by William J. Buehler, metallurgist at the U.S. Naval Ordinance Laboratory, through the fusion of 55% Nickel with 45% Titanium, leading to the creation of an alloy with peculiar characteristics of superelasticity and shape memory, that initially saw its only use in naval and military fields, but then it was, in a far-sighted way, also employed in the medical field, particularly in orthodontics and endodontics [23,24].

The success behind the NiTi alloy resides in its biological and mechanical properties that make the NiTi alloy unique and particularly suitable for the manufacturing of endodontic rotary instruments: biocompatibility [25], corrosion resistance [23,26], shape memory effect and superelasticity.

**Figure 1:**
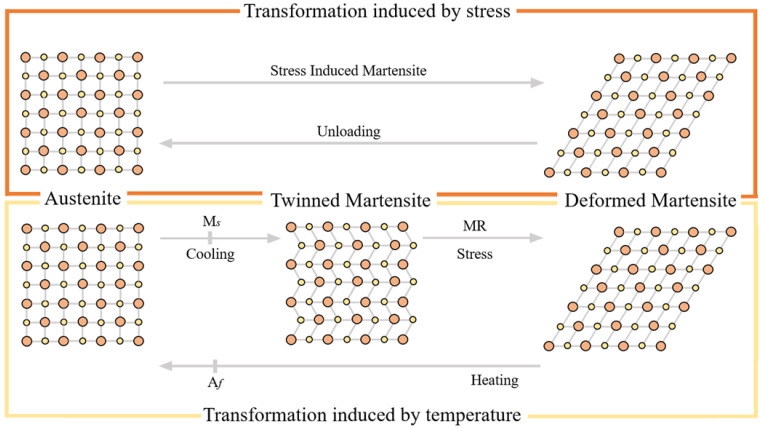
Schematic representation of phase crystallographic transformation of NiTi alloy induced by stress or temperature variation. Austenite has a body-centred cubic crystal lattice, whilst martensite can be divided in two different forms with different crystallographic organization: the twinned martensite that forms the structure of a closely packed hexagonal lattice and the deformed martensite or the detwinned martensite when there is a ‘flipping over’ type of shear [27].

The last two properties have a key role in determining the mechanical behaviour of NiTi rotary instruments. The first one, the shape memory effect, allows NiTi instruments to “memorize” a certain form and return by heating to its original form thanks to the phase transition between two crystallographic phases: a crystalline phase (called martensitic phase) which is stable below a certain critical temperature, and an austenitic phase that is stable above that critical temperature. The second one, the superelasticity, allows NiTi alloy, when mechanically loaded, to be reversibly deformed to very high strains (up to 8%) by the creation of a stress-induced phase, called Stress Induced Martensite (SIM). However, when the load is removed, the new SIM becomes unstable and the NiTi regains its original shape (Figure 1) [27,28,29].

**Figure 2:**
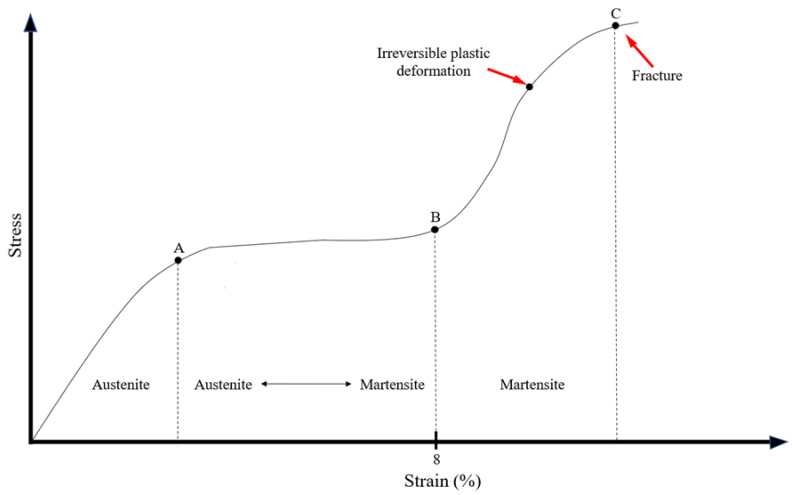
Schematic representation of the stress and strain curve showing the crystallographic transformation according to the induced stress.

The mechanical responses of the NiTi alloy under certain load can be represented through a stress/deformation graph (Figure 2). The stress and strain curve could be divided by three vertical line (A, B and C in Figure 2) that individuate on the graph three different areas according to the crystallographic organization of the NiTi alloy: the austenitic region in which the alloy is composed by austenite; the austenitic/martensitic region (also called R-phase) in which there is a partially transformation of austenite in martensite, according to the application of stress; the martensite region in which the total amount of austenite is transformed in martensite above certain loads [27,30]. Below certain load, the transformation induced by mechanical stress is totally reversible (elastic deformation) as a direct consequence of the superelasticiy, however if a yield strength is exceeded the deformation becomes irreversible (plastic deformation) and the endodontic instrument is permanently damaged [31].

Nevertheless, NiTi endodontic instruments, when subjected to stress that exceeds the limits of elasticity of the material, undergo plastic transformations and then fracture, if the stress is not removed. However, these limit values are much higher than those of the SS instruments of the same size and shape [1].

Reassuming, the transformation between different crystallographic phase could arise from the application of stress, the variation of temperature or a combination of these two factors. According to that, the NiTi alloy can exist in the solid state in three different crystalline phases, that differ between them for the three-dimensional atomic organization and for the mechanical properties: austenitic, martensitic and intermediate R phases [27].

**Figure 3:**
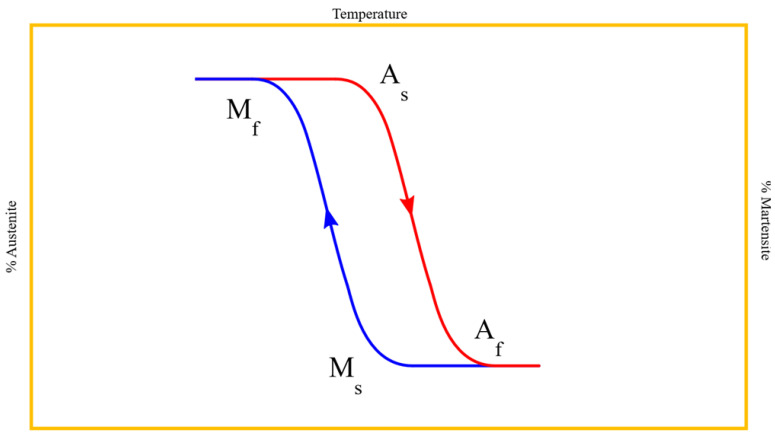
Diagrammatic representation of the temperature hysteresis diagram of NiTi alloy. (Ms) martensite start temperature, (Mf) martensite finish temperature, (As) austenite start temperature, (Af) austenite finish temperature. Since TTR strongly depends on different parameters such as blank wire, heat-treatment, manufacturing procedures and design, defining universal As, Af, Ms and Mf is almost impossible. However, for example, considering martensitic NiTi instruments the As temperature could range from 25 °C to 45 °C and Af temperature from 35 °C to 55 °C, whilst Ms temperature could range from 20 °C to 50 °C and Mf temperature from −25 °C to −35 °C. Regarding conventional NiTi instruments the As temperature could range from −30 °C to 5 °C and Af temperature from 5 °C to 20 °C, whilst Ms temperature could range from 20 °C to 10 °C and Mf temperature from −5 °C to −20 °C [32,34].

In the austenitic phase the NiTi alloy is characterized by a crystal structure with a body-centered cubic lattice that makes it stable at high temperatures and gives the alloy rigidity (high modulus of elasticity) and superelasticity, with very low plasticity characteristics. On the contrary, through a decrease of the temperature below the critical transformation temperature range (TTR) the NiTi alloy changes its crystallographic phase in the twinned martensitic phase, characterized by a closely packed hexagonal lattice (Figure 3). In this form the NiTi alloy shows stability at lower temperature with greater flexibility, cyclic fatigue resistance, softness and ductility, and exhibits the shape memory effect when heated [32,33]. Instead, the intermediate R-phase does not have a precise crystalline conformation but is rather a hybrid between the two phases described above. It is characterized by the possibility of its atoms to move between adjacent crystalline planes, continuously oscillating between an austenitic and martensitic organization.

As mentioned above, the martensite is not stable at intracanal temperature, so the conventional NiTi alloy is in the austenitic configuration, stable at high temperature. However, a change in the transformation temperatures (martensite start temperature (Ms), martensite finish temperature (Mf), austenite start temperature (As), austenite finish temperature (Af)) can be induced by thermal and mechanical treatments in such a way that endodontic instruments could be martensitic at the room temperature (Figure 3) [34,35]. With the aim of examining the TTR the most common method is the differential scanning calorimetry (DSC) analysis, that allows the evaluation of the transformation temperature trough controlled cooling and heating process [30]. However, sometimes the results of DSC analyses of NiTi rotary instruments are inaccurate due to the small dimensions of the endodontic instruments. For this reason, other analysis could be needed to confirm the results of DSC, such as XRD, micro-XRD, metallographic examination and scanning electron microscopy (SEM) observations [30].

Over the years, different thermal treatments have been proposed by manufacturers with the aim to obtain NiTi endodontic instruments with ever better mechanical performance (Table 1) [35].

### 1.3. Evaluation of Mechanical Properties of NiTi Endodontic Rotary Instruments

As described above, the introduction of Nickel-Titanium as the alloy of choice for the manufacturing of endodontic instruments has significantly revolutionized the shaping procedure and, thus, the root canal treatments. Nevertheless, the intracanal separation of endodontic rotary instruments remains one of the most concerns for endodontists. However, it has been demonstrated that the presence of a fractured instrument in itself does not definitely compromise the outcome of endodontic therapy, but surely requires a large investment of time and resources [36].

As reported by Sattapan et al., the two main causes of intracanal separation of NiTi endodontics instruments are cyclic fatigue and torsional stresses [37].

#### 1.3.1. Cyclic Fatigue Resistance

**Figure 4:**
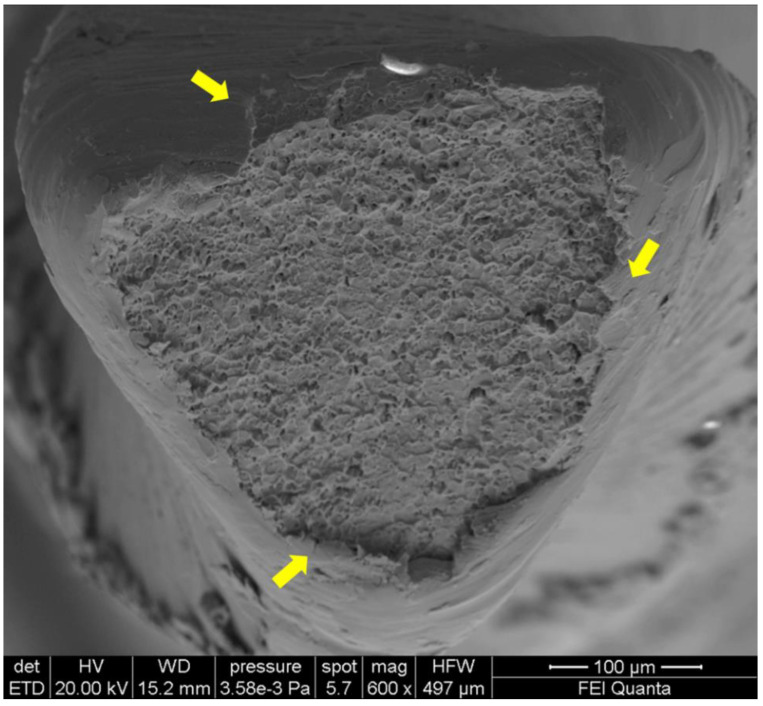
SEM image of fractured surface of a F2 EdgeTaper Platinum (Albuquerque, NM, USA) in a transversal view at ×600 magnification after cyclic fatigue testing. Dimples and microvoids visibly spread on the fractured surface constitute a typical feature of ductile fracture, which origins from the external part of the instruments with visible crack (evidenced by yellow arrows).

The cyclic fatigue accumulation is an unavoidable consequence of tension–compression strain cycles to which the instrument is subjected in the point of maximum curvature (Figure 4) [38]. As a result, the risk of fracture due to flexural fatigue can never be zeroed, but only limited, until these instruments are used in continuous or alternating rotation in curved canals.

With the aim of reducing the probability of intracanal failure arising from cyclic or flexural fatigue, the manufacturers and researchers have increasingly focused on the determination of those parameters that are directly or indirectly involved in the determination of cyclic fatigue resistance and flexibility of endodontic rotary instruments. In a general view, those parameters could be divided into three groups: the anatomy-related factors, the instrument-related factors and the factors related to the instrumentation technique and strategy.

The first group is composed by those factors that characterize the anatomy of the root canal system. Pruett et al. in 1997 proposed a new method for the evaluation of the complexity of the root canal anatomy, adding to the Schneider’s method another parameter: the radius of curvature of the root canal [38]. It has been demonstrated that in case of the same degrees of curvature, a smaller radius of curvature greatly reduces the resistance to cyclic fatigue of endodontic instruments. Accordingly, increasing the curvature angle and reducing the radius of curvature, endodontic instruments will be subjected to greater flexural stress, reducing their ability to withstand cyclic fatigue [38]. Thus, in order to prevent cyclic fatigue failure, the knowledge of the root canal anatomy is a fundamental prerogative [39,40].

Regarding the instrument-related factors, two parameters in particular must be mentioned: the heat-treatments, as described above, and the metal mass or the volume per millimeters (Vol per mm) [41,42,43]. Grande et al. have stated that there is a statistically significant relationship between the Vol per mm and the cyclic fatigue resistance of endodontic instruments, and that instruments with similar Vol per mm, and then mass, at the point of maximum stress show similar cyclic fatigue resistance. This innovative parameter, according to the Authors, allows to group in a single parameter all those geometric characteristics that, until then, were thought to have a crucial role in the determination of the cyclic fatigue resistance such as the number of blades, the size of the instrument, the taper and the inner core area [44].

The factors related to the instrumentation technique and the strategy used are mainly related to the access cavity design, the choice of the setting of speed and the motion used (continuous or reciprocating motion). As stated by Pedullà et al. a conservative access cavity could lead to an angled insertion of endodontic instruments inside the root canal system and the consequent decrease of their cyclic fatigue resistance arising from the increase of the flexural stress derived from their angulation of insertion [45]. As regards the use of endodontic instruments, it has been demonstrated that reducing the rotational speed, the time before fracture increases due to the lower number of cycles carried out in the same given time period, however, the rotational speed per se does not affect the number of rotations to fracture [46]. Finally, it has been demonstrated that the use of reciprocating movements (alternating clockwise and counterclockwise rotation movements) significantly reduces the cyclic fatigue of the instruments, increasing their resistance [47,48].

#### 1.3.2. Torsional Resistance

**Figure 5:**
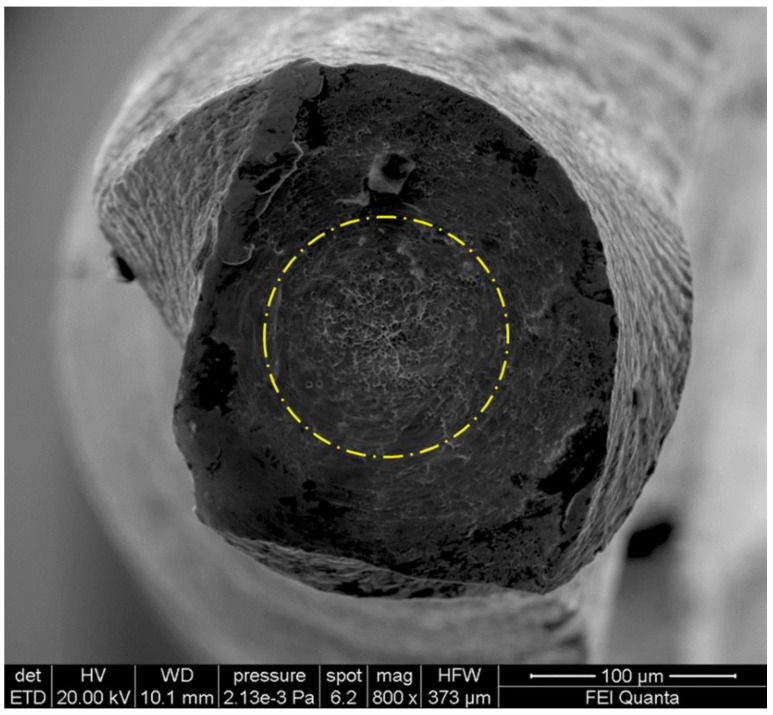
SEM image of fractured surface of a F-One #20 (Fanta Dental, Shanghai, China) in a transversal view at ×800 magnification after torsional testing. The typical features of fracture arising from excessive torsional load, showing concentric circular abrasion marks and fibrous dimples near the center of rotation are evidenced by the round-shaped circumferential line.

Torsional failure occurs when an apical part of the endodontic instrument, more frequently the tip, operating in continuous or alternating rotation, remains blocked in the dentinal wall and its coronal portion continues to rotate, causing its fracture (Figure 5) [37].

Even the parameters that influence the torsional resistance of NiTi endodontic rotary instruments could be divided in the same three groups evidenced in reference to the cyclic fatigue resistance [49].

The anatomy related factors include all the characteristics of the tooth that contribute to the generation of torsional stresses such as: the diameter of the canal, the radius and degrees of curvature, the hardness of the dentin and the length of the canal [50,51,52].

Otherwise, in the third group there are all those factors that characterize the clinical approach to endodontic therapy such as: the extension of the access cavity, the coronal preflaring, the glide path, the use of high and low torque engines, the instrumentation technique (crown-down, step back, simultaneous instrumentation technique, etc.), the amplitude and intensity of the pecking-motion and the type of motion used (reciprocating or continuous motion) [53,54,55,56,57,58,59]. Contrarily to the cyclic fatigue resistance, a constricted access cavity improves the torsional resistance of endodontic instruments [60]. In fact, it has been stated that increasing the bending moment acting on an endodontic instrument, as it happens during the angled insertion of the instruments in the conservative access cavity, the torsional resistance increases [60,61]. In other words, according to Di Nardo et al., constricted access cavity could impose a coronal curvature to NiTi instruments, increasing the bending moment acting on them [60]. An increased bending moment, according to Seracchiani et al. is able to increase the torsional resistance of NiTi instruments since the two parameters are strictly correlated as discussed in the next subparagraph [61].

Finally, the second group consists of all those structural factors that characterize an endodontic instrument, such as the cross-sectional design, the type of the alloy and its crystallography phase, the manufacturing processes, the pitch, the helix and the rake angle, the length of the instrument and the taper [49,55,62,63,64]. As many studies have shown, the design of the cross-section is one of the most important parameters that can significantly determine the torsional stiffness of a NiTi endodontic instrument, since it thoroughly influences its mechanical properties [62,65,66]. Berutti et al. have been demonstrated that different cross-sectional designs allow a different distribution of torsional stresses; the more stresses are uniformly spread along the instrument, the more its torsional stiffness increases [67]. Nevertheless, the actual relationship between the cross-sectional design of the instrument and its torsional stiffness seemed to be unclear and, specifically, it was unclear which aspect of the instrument cross-section played a major role in determining the resistance of the instruments to torsional stresses. Recently, Zanza et al. identified the key factor in determining the torsional resistance of NiTi endodontic rotary instruments. Based on their study, the parameter that showed the most significant correlation with torsional resistance is the polar moment of inertia [68]. According to this, the Authors stated that the mass and area are not so crucial in terms of absolute value, but instead, it is relevant how they are distributed in relation to the center of rotation. Thus, the more the mass and the area are spread far from the pivot center, the more the polar moment of inertia is and the more the torsional resistance is [68].

#### 1.3.3. Combined Torsional and Flexural Stresses

Recently, greater attention has been focused on understanding the interaction between bending and torsional stress in order to better comprise the mechanical phenomena behind the root canal instrumentation [61,69]. In fact, the NiTi endodontic rotary instruments during shaping procedure are always subjected to both flexural and torsional stresses and surely further studies are needed to eviscerate their reciprocal influence in a more detail [70,71].

Seracchiani et al. concluded that in static situation flexural stresses significantly influence the torsional resistance of instruments with a blocked tip. This is due to the fact that the torque, in case of curved canal, is not only caused by torsional moment, but also by flexural loads [61]. Thus, it can be stated that increasing the curvature degrees of root canal the torsional resistance of instruments increases.

On the contrary, Iacono et al. with the aim of investigating the influence of torsional loads on cyclic fatigue of NiTi endodontic instruments proposed a novel testing device [69]. The device is a usual cyclic fatigue testing device with a standardized load on the apical 5 mm providing a uniform real-time load. The Authors concluded that an increase of apical torsional load led to a decrease of cyclic fatigue resistance [69]. However, even if the above-mentioned research add novelty to the current knowledge, the influence of torsional loads on cyclic fatigue in the point of maximum curvature, where the bending moment acts, is still unknow. Therefore, further research should be conducted on this theme.

Another limitation on this topic is the static condition used in the methodology of the published research. In fact, in order to comprise in a more detail the reciprocal relationship between cyclic fatigue and torsional resistance, a dynamic evaluation of these phenomena is mandatory [72].

#### 1.3.4. Bending Ability

The flexibility of NiTi endodontic rotary instruments is defined as the ability to be bent without being irreversibly deformed and still retaining their original form [73]. As previously stated by several studies, the enhanced flexibility in comparison to SS manual instruments arises from the NiTi alloy superelasticity and the ability to start a stress-induced transformation of the parent β-phase, characterized by the reversible transition of austenite to martensite [73,74,75]. Moreover, the increased bending ability of NiTi alloy is highlighted by the NiTi Young’s modulus (modulus of elasticity), an intrinsic characteristic of the alloy, that is lower than the stainless-steel one [1].

As demonstrated, an increased flexibility of NiTi endodontic instruments allows a more suitable canal enlargement since the instrument is more able to follow the curved anatomy of root canals and to maintain a central position within the canal [1,5,76,77,78]. Flexibility is influenced by several factors, among those the most important is undoubtedly the heat-treatment that allows instrument to be martensitic at ambient or intracanal temperature [35]. However, there are other factors related to the bending ability of NiTi instruments, such as the alloys chemical composition, the geometric design such as cross-section, inner core area, taper and pitch [30,79,80].

According to the bending test assessed by ISO 3630-1, the flexibility of an endodontic instrument is evaluated by clamping 3 mm of its tip in a chuck and applying an angular deflection of 45°. The force generated to bend the instrument is registered as the bending resistance, thus, low bending results are indicative of the high material flexibility [81].

Recently, Miccoli et al. proposed a new bending test device able to evaluate the flexibility of NiTi instruments at different length from the tip (i.e., 3, 6 and 9 mm), providing a more representative description of the bending ability of NiTi instruments [82].

The main limitation of bending tests is always the staticity of the evaluation. In fact, those tests do not take into account the dynamicity of instrumentation procedures, not considering the rotation of NiTi instruments at high speed, thus, the clinical relevance of these static tests has been considered low by many researchers, since clinical usage can be affected by several other factors [83]. Despite this, static bending tests with static torsional and cyclic fatigue tests remain a good manner to establish the basic mechanical properties of NiTi instruments, that should be implemented with dynamic investigations such as the evaluation of centering ability, canal transportation, shaping ability and cutting efficiency, so a reliable evaluation of the performance of different NiTi instruments can be performed through a multimethod approach [83].

### 1.4. Centering Ability, Canal Transportation and Shaping Ability

The main goal of shaping procedure is undoubtedly the mechanical removal of vital and/or necrotic tissues form the root canal system, simultaneously allowing the creation of an adequate space for the chemical disinfection and obturation [84]. According to this, root canal instrumentation could be considered as the most crucial phase during root canal treatment, in which clinicians must avoid any procedural error in order to not compromise the outcome of the endodontic treatment [85,86]. As stated by Gorni et al. the alteration of the root canal morphology is one of the most significant parameters in determining the outcome of endodontic retreatments, since inferior cleansing can be performed specifically aimed at the anatomical irregularities created by previous treatment [87]. Regarding this, the most common procedural errors during root canal treatments could be synthetized in: ledges, strip perforations, excessive thinning of canal walls, and canal transportation [1,88,89].

Considering the above-mentioned reasons, the preservation of the original root canal morphology is one of the most important features that characterized endodontic instruments. In fact, during the evaluation of the performance of NiTi rotary instruments the shaping ability should be considered. As stated by several Authors, the shaping ability could be considered as a macro-group of characteristics that comprises centering ability, canal transportation, difference between canal volume before and after therapy and the ratio between touched an untouched dentinal area [90,91,92,93,94,95].

The two main popular and thoroughly validated methods used to evaluate these factors are the Micro-Computed Tomography (CT) and the SEM analysis, singularly used or in combination, that allow a precise calculation of the interested measurement trough the aid of digital software [90,92]. In our opinion Micro-CT imaging should be preferred because it is non-destructive 3-dimensional analysis and gives high-resolution images to precisely evaluate the untouched area, volume changes, and transportation in comparison to SEM analysis that requires the split of the teeth [90]. However, the SEM analysis could be used to evaluate debris and smear layer removal since it allows their direct measurement without using complex software for the voxel interpretation of Micro-CT images, nevertheless, also those measurements have some limitation, such as the bi-dimensional analysis of debris and smear layer, that does not allow measurement of the thickness of both parameters analyzed [96,97,98].

The most widely used centering ability and canal transportation evaluation method is the superimposition of root canal anatomy images before and after instrumentation [16,90,99,100]. The differences reside in the acquisition method used. Obviously the most accurate is the Micro-CT, followed by the CBCT and bi-dimensional radiograph. During Micro-Ct analysis, scans of each specimen before and after shaping procedures are overlapped using algorithms, allowing a consistent location of various dimensional measurements, such as the measurement of transportation across different (pre- and post-shaping) CT scans [95].

According to Silva et al., the shaping ability can be evaluated measuring the untouched canal area and the quantification of accumulated hard-tissue debris. Using the Micro-CT analysis, the first one is calculated as a percentage of the number of static voxels (voxels present in the exact same position on the canal surface before and after instrumentation) using the following formula [101,102]:

(Number of static voxels × 100): (total number of surface voxels).

The latter, instead, is calculated as the percentage volume of the original canal morphology after intersecting the stacks of sound and instrumented root canal system [103], where material with density similar to dentine inside instrumented canal regions, which were previously occupied by air, was considered debris [104,105].

As pointed out by several studies, the parameters that influence the centering ability, canal transportation and shaping ability of NiTi endodontic instruments are fundamentally related to the morphology (cross section, pitch, taper and helix angle), heat-treatment and instrumentation procedures (speed and motion) [99,106]. Htun et al. stated that the Gentlefile (GF; MedicNRG, Kibbutz Afikim, Israel) is able to achieve great results in terms of superior smear layer removal, less remaining debris and canal transportation in comparison to other NiTi instruments because of its ultraflexibility, high rotational speed, and shaping by abrading [92]. In addition, Velozo et al. in a review of the literature pointed out that XP-endo^®^ Shaper instrument (FKG Dentaire, La Chaux-de-Fonds, Switzerland) in comparison to other NiTi systems showed greater shaping ability because of the possibility to use it at higher speed and its morphology, characterized by booster tip with an asymmetrical rotation that guarantee an expansion [107].

Despite the recent advances and innovations in kinematics and metallurgical and mechanical characteristics, none of the NiTi instrument systems are capable of shaping root canals to the ideal form, leaving a certain percentage of untouched canal [108,109,110].

## 2. Materials and Methods

A search for articles on the mechanical characteristics, testing and future perspective was performed using PubMed electronic database.

In this case, 458 articles were screened, and only 98 studies were included, exclusively for the purpose of analyzing the mechanical characteristics of these instruments.

According to the authors, only some articles about NiTi rotary instruments which best represented the aim of this study were selected.

The only articles selected and not related to these intentions previously set out were considered only for the technical specifications and considerations on the ownership of the NiTi instruments.

All research articles concerning NiTi rotary files that did not provide significant indications about the mechanical, physical and application properties of this instruments, or for its improvement, were excluded from this study.

Furthermore, the article that more than others represented the indication for the daily use of this technology and for its future development, was included in this analysis.

## 3. Results

As established, the improvements introduced in the endodontic technique by these instruments are extremely relevant, and here summarized in their main characteristics:

- Reduction in time of endodontic treatments,
- Simplification of instrumentation procedures,
- Increase of predictability and effectiveness of endodontic treatments.

### 3.1. Thermal Treatments

Over the years, different thermal treatments have been proposed by manufacturers with the aim to obtain NiTi endodontic instruments with ever better mechanical performance, with excellent clinical results. In fact, it is reported that, with the same shape characteristics, different thermal treatments on NiTi endodontic instruments seem to have a considerable influence on the resistance of the instruments if subjected to different stresses [42,43,44,52,61].

### 3.2. Cyclic Fatigue and Torsional Stress Resistance

The two main causes of intracanal separation of NiTi endodontics instruments are cyclic fatigue and torsional stresses, and on these over the years new instruments have been developed that are able to withstand increasingly considerable stresses.

Regarding cyclic fatigue and the instrument-related factors, two parameters in particular must be mentioned: the heat-treatments, as described above, and the metal mass or the volume per millimeters (Vol per mm) [41,42,43]. This innovative parameter, according to the Authors, allows to group in a single parameter all those geometric characteristics that, until then, were thought to have a crucial role in the determination of the cyclic fatigue resistance such as the number of blades, the size of the instrument, the taper and the inner core area. The factors related to the instrumentation technique and the strategy used are mainly related to the access cavity design, the choice of the setting of speed and the motion used (continuous or reciprocating motion). A conservative access cavity could lead to an angled insertion of endodontic instruments inside the root canal system and the consequent decrease of their cyclic fatigue resistance arising from the increase of the flexural stress derived from their angulation of insertion. It has been demonstrated that reducing the rotational speed, the time before fracture increases due to the lower number of cycles carried out in the same given time period, however, the rotational speed per se does not affect the number of rotations to fracture [45,46,47].

Regarding torsional stress resistance, this is mainly influenced by the extension of the access cavity, the coronal preflaring, the glide path, the use of high and low torque engines, the instrumentation technique (crown-down, step back, simultaneous instrumentation technique, etc.), the amplitude and intensity of the pecking-motion and the type of motion used (reciprocating or continuous motion) [65,66,67,68]. Since it is still unclear which aspect of the instrument cross-section played a major role in determining the resistance of the instruments to torsional stresses, recently, some Authors identified the key factor in determining the torsional resistance of NiTi endodontic rotary instruments [68]. Based on their study, the parameter that showed the most significant correlation with torsional resistance is the polar moment of inertia. Some Authors concluded that in static situation flexural stresses significantly influence the torsional resistance of instruments with a blocked tip [61]. On the contrary, other Authors concluded that an increase of apical torsional load led to a decrease of cyclic fatigue resistance [69].

### 3.3. Bending Tests

Although to date dynamic tests are conventionally considered the best for the complex and comparative evaluation of the characteristics of the different instruments, static bending tests with static torsional and cyclic fatigue tests remain a good manner to establish the basic mechanical properties of NiTi instruments, that should be implemented with dynamic investigations such as the evaluation of centering ability, canal transportation, shaping ability and cutting efficiency, so a reliable evaluation of the performance of different NiTi instruments can be performed through a multimethod approach [83].

### 3.4. Centering Ability

As pointed out by several studies, the parameters that influence the centering ability, canal transportation and shaping ability of NiTi endodontic instruments are fundamentally related to the morphology (cross section, pitch, taper and helix angle), heat-treatment and instrumentation procedures (speed and motion) [99,106].

## 4. Discussion

The main drawback of several studies is the limited analysis performed for the comparison of NiTi systems. In other words, most of research are focalized on the static characteristics of instruments such as static cyclic fatigue, torsional loads and flexibility, not considering their reciprocal interaction and all dynamic factors that, in our opinion, are the main parameters that characterized endodontic systems [111]. Obviously, static mechanical behavior is fundamental to assess the basic performance of NiTi instruments, but its interpretation during clinical practice is pointless since it does not resemble the real clinical scenario [83]. Results arising from static tests should not be interpreted as isolated factors but should be considered in a general and more complete view. As mentioned before, the main goal of instrumentation is to establish an adequate root canal volume that allows chemical disinfection and obturation. Thus, flexibility and torsional resistance are only secondary parameters if shaping ability is not considered. For those reasons, as stated by Silva et al., multimethod research that investigate both static and dynamic performance of NiTi instruments should be strongly recommended in order to give to original research an actual clinical significance [83].

Regarding this, we strongly encourage researcher to make effort to stipulate novel methodology that allow a complete comprehension of mechanical and metallurgical behavior of NiTi rotary instruments in order to achieve significant comparison between different NiTi systems.

## 5. Conclusions

The knowledge of the properties of NiTi alloy and an in-depth understanding of the mechanical behavior of endodontic instruments during instrumentation procedure is mandatory in order to improve techniques, instrument design and their clinical use. Intracanal separation of NiTi endodontic rotary instruments is still one of the major concerns of endodontists, even if the success rate of root canal therapy is high. Reducing the percentage of instrument failure is a fundamental future perspective and a deep comprehension of The NiTi alloy and stresses acting on the endodontic instruments during shaping procedures are needed to achieve this goal.

## Figures and Tables

**Table 1:** Overview of NiTi alloy used for the manufacture of endodontic instruments.

Alloy	Crystallographic Phase	NiTi System
*Conventional NiTi alloy*	Austenitic	Mtwo OneShape ProFile ProTaper Universal RaCe, BioRaCe, iRace F360, F6 Skytaper EdgeTaper
*R-phase*	Austenitic	Twisted File Twisted File Adaptive K3XF (not twisted)
*M-Wire*	Austenitic with small amounts of R-phase and martensite	ProFile Vortex ProFile GT Series X ProTaper Next Reciproc WaveOne
*CM Wire*	Martensitic with varying amounts of austenite and R-phase	Hyflex CM THYPOON Infinite Flex NiTi Files V-Taper 2H Hyflex EDM
*Gold heat-treated*	Martensitic with varying amounts of austenite and R-phase	ProTaper Gold WaveOne Gold
*Blue heat-treated*	Martensitic with varying amounts of austenite and R-phase	ProFile Vortex Blue Reciproc Blue Rotate
*MaxWire*	Martensitic (20 °C), austenitic (35 °C)	XP-endo Finisher XP-endo Shaper
*T-Wire*	Martensitic with varying amounts of austenite and R-phase	2Shape
*C-Wire*	Martensitic with varying amounts of austenite and R-phase	OneCurve RECI One
*FireWire*	Martensitic with varying amounts of austenite and R-phase	EdgeOne Fire EdgeSequel Sapphire EdgeTaper Platinum EdgeFile X7
*AF-R Wire*	Martensitic with varying amounts of austenite and R-phase	AF-One
*FKG heat treatment*	Phase transition ranging from 32 °C to 35 °C (between martensite and austenite)	R-Motion file System Race Evo

## Data Availability

The data presented in this study are available on request from the corresponding author.

## Abbreviations

NiTi: Nickel-Titanium; SS: Stainless-Steel; SIM:Stress Induced Martensite; TTR: Transformation Temperature Range; Ms: Martensite Start Temperature; Mf: Martensite Finish Temperature; As: Austenite Start Temperature; Af: Austenite Finish Temperature; DSC: Differential Scanning Calorimetry; SEM: Scanning Electron Microscopy; Vol per mm: Volume per Millimetres (Vol per mm).
